# Activated Human Memory B Lymphocytes Use CR4 (CD11c/CD18) for Adhesion, Migration, and Proliferation

**DOI:** 10.3389/fimmu.2020.565458

**Published:** 2020-09-29

**Authors:** Zsuzsa Nagy-Baló, Richárd Kiss, Alina Menge, Csaba Bödör, Zsuzsa Bajtay, Anna Erdei

**Affiliations:** ^1^Department of Immunology, Eötvös Loránd University, Budapest, Hungary; ^2^MTA-SE Momentum Molecular Oncohematology Research Group, First Department of Pathology and Experimental Cancer Research, Semmelweis University, Budapest, Hungary; ^3^MTA-ELTE Immunology Research Group, Eötvös Loránd University, Budapest, Hungary

**Keywords:** human memory B cells, CR4 (CD11c/CD18), β2 integrin, adhesion, migration, proliferation

## Abstract

Complement receptors CR3 (CD11b/CD18) and CR4 (CD11c/CD18) of myeloid cells are known for long to participate in actin linked functions like phagocytosis, adhesion, and migration. The expression and role of these two β_2_-integrins however, in human B lymphocytes have only scarcely been studied so far, although it has been shown recently that CD11c^+^ B cells are mainly memory cells. In our systematic study we investigated B cells isolated from tonsils and peripheral blood of healthy donors. We found, that while only 5% of resting tonsillar B cells expressed CD11c, their number increased up to 26% after 3 days of BCR stimulation. Lower, but still remarkable percentage of B lymphocytes were positive for CD11c after stimulation via TLR9 alone or via TLR9 and BCR simultaneously. At the same time, we detected no significant expression of CD11b on resting or activated tonsillar B cells. Blood B lymphocytes showed a similar expression pattern of both β_2_-integrins. We demonstrated that CD11c molecules appearing on the surface of B cells are newly synthesized, reaching the number of 9,500 per activated B cell. We found that CR4 expressing B cells belong to the memory pool and the increase of CD11c expression on tonsillar B cells upon BCR mediated activation occurs parallel with class switching. Analysis of the function of CD11c revealed, that this β_2_-integrin contributes to the adhesion and migration of activated B lymphocytes. We also demonstrated that the CR4 mediated adhesion promotes the proliferation of the BCR activated cells. Our studies are the first to demonstrate that CD11c expressed on BCR-activated human B cells are not only passive markers but functional drivers of memory B cell responses.

## Introduction

Integrins mediate versatile functions that are associated with cell-cell and cell-extracellular matrix interactions, controlling the response to different environmental stimuli. They serve as a bridge between the extracellular matrix and the intracellular cytoskeletal network by mediating actin-linked functions like adhesion or migration. Integrins expressed on the cell membrane are mostly in their inactive state, which allows free circulation of leukocytes in blood vessels. Their activation is conformationally regulated and can be induced by intracellular and extracellular stimuli leading to a “switchblade-like” conformational change. These result in their active, extended state in which they are able to bind their ligands and fulfill various functions (1).

Complement receptors CR3 (CD11b/CD18, also known as Mac-1, α_M_β_2_) and CR4 (CD11c/CD18, also designated as p150,95; α_X_β_2_) are members of the β_2_ integrin family. While they contain the same β chain and the extracellular region of their α chain is very similar (showing 87% homology), the cytoplasmic tail of CD11b and CD11c has a highly different structure. This domain of CD11b is one-third shorter than the corresponding part of CD11c and shares only 56% homology with it. This variance leads to functional differences between CR3 and CR4, as the kinetics of activation of β_2_ integrins and their ability to associate with signaling or actin-binding proteins depend on their cytoplasmic domain, including the intracellular tail of the α chain (2–4).

CR3 and CR4 bind multiple ligands like iC3b, ICAM-1 and fibrinogen and have well-known functions in human myeloid cells including phagocytosis, adhesion and migration (1). While CR3 and CR4 have been generally thought to mediate overlapping functions, we have previously shown on human myeloid cells that CR3 is more important in iC3b mediated phagocytosis (5), while CR4 has a dominant role in adhesion to fibrinogen (6).

In contrast to the ever-growing information of their role in myeloid cell types, our knowledge about the expression and function of CR3 and CR4 on human B lymphocytes is limited. Though these two receptors are not widely expressed on non-activated B lymphocytes of healthy donors (7, 8), they can be detected on certain B cell populations or after activation by different stimuli (7, 9–13). Regarding their function, CR3 was shown to contribute to the high migratory potential of the CD11b^+^ B lymphocytes, potentially helping inflammatory processes (12, 14), while CR4 was demonstrated to be involved in the adhesion and proliferation of PMA stimulated B cells (7). Recently, Golinski et al. demonstrated that CD11c^+^ B cells differentiate into antibody-secreting cells upon activation, however the role of CR4 in this process was not clarified (13).

The aim of the present study was to gain a better knowledge of the distribution and role of CR3 and CR4 of human B lymphocytes obtained from blood and tonsil. So far mostly peripheral B cells were studied, although B cell activation and maturation take place in the secondary lymphoid organs—such as tonsils. Thus, a deeper insight into the expression and function of CR3 and CR4 of tonsillar B lymphocytes is needed to get a better understanding of the physiological role of these β_2_ integrins. It is also important to point out that in contrast to former studies where human B cells were stimulated with the non-physiological phorbol myristate acetate (PMA), we used B-cell receptor (BCR) activated cells to investigate the expression and role of the β_2_ integrins. We found that none of these two receptors were present in high percentages of non-stimulated human B cells, after 3 days of activation however, on average 26% of B cells expressed CD11c, but not CD11b. Beside many other ligands CR4 can bind fibrinogen as well, which was shown to be present in high amounts on follicular dendritic cells in the dark zone of GCs of human tonsils, moreover, it was shown to stimulate the proliferation and survival of the BCR stimulated L3055 cell line (15). Since we found that the vast majority of CD11c positive B cells belong to the switched memory pool, we focused on the role of fibrinogen and studied the role of this ligand on various functions of primary B cells activated via their BCR. We prove that CR4 on BCR activated tonsillar B lymphocytes is not merely a passive marker, but it contributes to the adhesion and migration of the cells, furthermore, CR4 mediated adhesion enhances the proliferation of memory B lymphocytes.

## Materials and Methods

### Isolation of B Cells

Tonsils were obtained from children undergoing routine tonsillectomy in the Central Hospital of Southern Pest National Institute of Hematology and Infectious Diseases in Budapest, Hungary, in accordance with the Helsinki Declaration and was approved by the Ethics Committee of the Medical Research Council in Hungary (TUKEB), 52088/2015/EKU. After mechanical destruction of the tonsillar tissue, mononuclear cells were isolated by Ficoll-Hypaque gradient centrifugation (GE Healthcare, Chicago, IL, USA). T lymphocytes were rosetted by the addition of 2-aminoethylisothiouronium bromide (Sigma-Aldrich, St. Louis, MO, USA) treated sheep red blood cells, and B cells were isolated by a second centrifugation over Ficoll-Hypaque solution. For each experiment, high-density (“resting”) B cells were used, separated on Percoll (Sigma-Aldrich, St. Louis, MO, USA) gradient. The purity of B cells was higher than 95% in each case, as measured by flow cytometry using FITC-conjugated mouse-anti-human CD19 antibody (clone LT19, ImmunoTools GmbH, Friesoythe, Germany). For the RQ-PCR measurements tonsillar B cell samples were further purified by sorting CD19 positive cells using a FACS Aria III instrument and the FACSDiva software, reaching a purity higher than 99%.

Peripheral blood B lymphocytes were isolated from buffy coat obtained from healthy donors and provided by the Hungarian National Blood Transfusion Service. Informed consent was provided for the use of blood samples according to the Helsinki Declaration. Peripheral blood mononuclear cells (PBMC) were separated by Ficoll-Hypaque gradient centrifugation and B cells were isolated by negative selection using the Pan B Cell Isolation Kit (Miltenyi Biotech, Bergisch Gladbach, Germany).

### Culture Conditions

Isolated tonsillar or blood B cells were cultured in RPMI-1640 medium (Sigma-Aldrich, St. Louis, MO, USA) containing 10% FCS (Thermo Scientific, Rockford, IL, USA) and 50 μg/ml gentamycin (Sigma-Aldrich) at 37°C and 5% CO_2_, and were activated using 5 μg/ml F(ab')_2_ anti-human IgG/A/M (Jackson ImmunoResearch, Cambridgeshire, UK) and/or 0.5 μg/ml CpG- ODN 2006 (Sigma-Aldrich, St. Louis, MO, USA) for 3 days as indicated.

### Flow Cytometry

Flow cytometry was carried out with resting B cells right after isolation and with stimulated cells at day 3 of culturing. To measure CD11b expression mouse anti-CD11b antibody (clone ICRF44, Biolegend, San Diego, CA, USA) with Alexa 647 conjugated anti-mouse antibody (Invitrogen, Thermo Fisher Scientific, Waltham, MA, USA) were used. To characterize CD11c positive B cells the following antibodies were used: PE-conjugated anti-CD11c (clone BU15, ImmunoTools GmbH, Friesoythe, Germany), APC-conjugated anti-CD27 (clone LT27, ImmunoTools) and biotin-conjugated anti-human IgD (BD Biosciences, San Jose, CA, USA) with Streptavidin, Alexa Fluor 488 conjugate (Invitrogen). We used an Alexa Fluor 488 conjugated antibody specific to the active conformation of CD18 (clone m24, Biolegend, San Diego, CA, USA) to verify that β2 integrins on BCR activated CD11c^+^ B cells switched to their active state under the conditions of the functional assays. To rule out dead cells from the analyses we used DAPI (Thermo Scientific, Rockford, IL, USA) staining.

The absolute number of CD11c was determined by using Qifikit (Dako, Agilent, Santa Clara, CA, USA) according to the manufacturers' instructions. Briefly, cells were labeled with saturating concentration of mouse-anti-human CD11c antibody (clone BU15, ImmunoTools) and with FITC-conjugated F(ab)'_2_ fragment goat anti-mouse secondary antibody (Jackson ImmunoResearch, Cambridgeshire, UK). Beads carrying defined amounts of mouse IgG were labeled with the same secondary antibody to specify the correlation between fluorescence intensity and the number of antibodies bound. The calibration curve established with the beads was used to determine the number of bound anti-CD11c antibodies on the surface of B lymphocytes.

Measurements were performed using a CytoFLEX cytometer (Beckman Coulter Life Sciences, Indianapolis, IN, USA) employing the CyteExpert software. Data were analyzed using the CytExpert and Kaluza softwares.

### RNA Isolation and Real-Time Quantitative PCR

B cells were collected immediately after sorting or following BCR mediated activation and total RNA was isolated using TRIzol Reagent (Invitrogen, Thermo Fisher Scientific) following the manufacturer's protocol. Hundred ng of the isolated RNA was reverse transcribed to cDNA using the High-Capacity cDNA Reverse Transcription Kit (Thermo Fisher Scientific, Waltham, MA, USA).

The real-time quantitative PCR (RQ-PCR) assay was performed with Quantstudio® 3 Real-Time PCR System (Thermo Fisher Scientific) and Quantstudio Design & Analysis Desktop Software version 1.4 (Thermo Fisher Scientific) was used to analyse the data after amplification. For amplification of CD11b and CD11c coding mRNA predesigned Taqman assays for *ITGAX* (Hs00174217_m1) and *ITGAM* (Hs01064805_m1) (Thermo Fisher) were used. RQ-PCR was performed in duplicates, for 40 cycles (95°C for 1 s, 60°C for 20 s), and the relative quantity of each mRNA was calculated applying the comparative C_T_ method using human *GUSB* (Hs99999908_m1, Thermo Fisher) endogenous control as reference gene.

### Studying the Role of CD11c in B Cell Functions

The measurement of adherence and the analysis of migration was performed on BCR-activated tonsillar B cells on the 3rd day of the cell culture. Before and during the assay cells were incubated with Fc-receptor blocking reagent (Miltenyi Biotec, Bergisch Gladbach, Germany) to avoid Fc-receptor mediated binding of the CD11c specific antibody. For blocking the function of CR4, cells were treated with 10 μg/ml CD11c-blocking antibody (mouse IgG1, clone BU15, ImmunoTools GmbH, Friesoythe, Germany) for 30 min at 4°C. As control, CD71 specific antibody (mouse IgG1, clone OKT9, Thermo Fisher Scientific, Waltham, MA, USA) was used. To ensure that integrins recycled from the cytoplasm are also blocked, the antibodies were not washed out for the assay. To strengthen the results obtained by using the CD11c blocking antibody, we also carried out the experiments employing CD11c^−^ cells only. To this end CD11c^+^ cells were depleted from the B cell pool using the CD11c specific antibody and Anti-Mouse IgG MicroBeads (Miltenyi Biotech, Bergisch Gladbach, Germany) according to the manufacturers' instructions.

### Measurement of Adherence

Ninety Six-well CELLview cell culture dish with glass bottom (Greiner Bio-One, Kremsmünster, Austria) was coated with 10 μg/ml fibrinogen (Merck, Budapest, Hungary) for 1 h at 37°C. After washing, non-specific binding sites were masked by adding 250 μg/ml synthetic copolymer PLL-*g*-PEG (ethylene glycol) (PLL-*g*-PEG, SuSoS AG, Dübendorf, Switzerland) for 1 h at 37°C. As negative control, cells were let to adhere to surfaces only blocked with PLL-*g*-PEG. Antibody treated cells were allowed to adhere to the fibrinogen-coated and/or PLL-PEG blocked surfaces for 1 h at 37°C and 5% CO_2_ in 100 μl of medium, in duplicates. After fixing with 2% paraformaldehyde (Sigma-Aldrich, St. Louis, MO, USA) for 10 min unbound cells were washed away and the adherent cells were stained with Draq5 (Biolegend, San Diego, CA, USA) and phalloidin-Alexa488 (Molecular Probes, Thermo Fisher Scientific, Waltham, MA, USA) containing 0.1% Triton X-100 (Reanal, Budapest, Hungary). Images were taken by Olympus IX81 laser scanning confocal microscope using the FluoView 500 software. Eight representative fields were scanned in two wells for each treatment and nuclei were counted using ImageJ software.

### Measurement of Migration

Analysis of migration has been performed using 24 well transwell plates (polycarbonate membrane with 5.0 μm pore, Corning, New York, NY, USA) after coating the membranes with 100 μg/ml fibrinogen overnight at 37°C and masking non-specific binding sites with 250 μg/ml PLL-*g*-PEG for 1 h at 37°C. B cells treated with the antibodies as described above were added to the upper chamber of the transwell plate in 100 μl RPMI-1640 medium, while the lower chamber contained 100 ng/ml SDF-1α (Thermo Fisher Scientific, Waltham, MA, USA). As negative controls the number of cells migrating through the transwell membrane only blocked with PLL-PEG and the number of cells migrating in the absence of the chemoattractant was counted. Cells were let to migrate for 4 h at 37°C, then the upper chamber was removed and 25 mM EDTA was added to the lower chamber. Transmigrated cells were collected from the lower chamber and counted immediately by a CytoFLEX cytometer (Beckman Coulter Life Sciences, Indianapolis, IN, USA) using the CytExpert software.

### Proliferation Assay

Freshly isolated high-density B cells were seeded onto 96-well flat bottom culture plates precoated with 10 μg/ml fibrinogen and/or blocked with 250 μg/ml PLL-*g*-PEG where indicated, in triplicates, at 2 × 10^5^ cells/well. Cells were stimulated with 5 μg/ml goat anti-human IgG/A/M F(ab')_2_ (Jackson ImmunoResearch, Cambridgeshire, UK) for 3 days. After 48 h cells were pulsed with 1 μCi/well ^3^H-thymidine (NEN, Boston, MA, USA) for 18 h. Incorporated radioactivity was measured with a Wallac 1409 liquid scintillation β counter (Wallac, Allerod, Denmark).

### Statistics

We compared each treatment to the appropriate control sample, presented on the graphs as 100%. Statistical tests were performed with GraphPad Prism 6 software, with *p* < 0.05 considered significant.

## Results

### Activated Human B Lymphocytes Express CR4 but Not CR3

**Figure 1:**
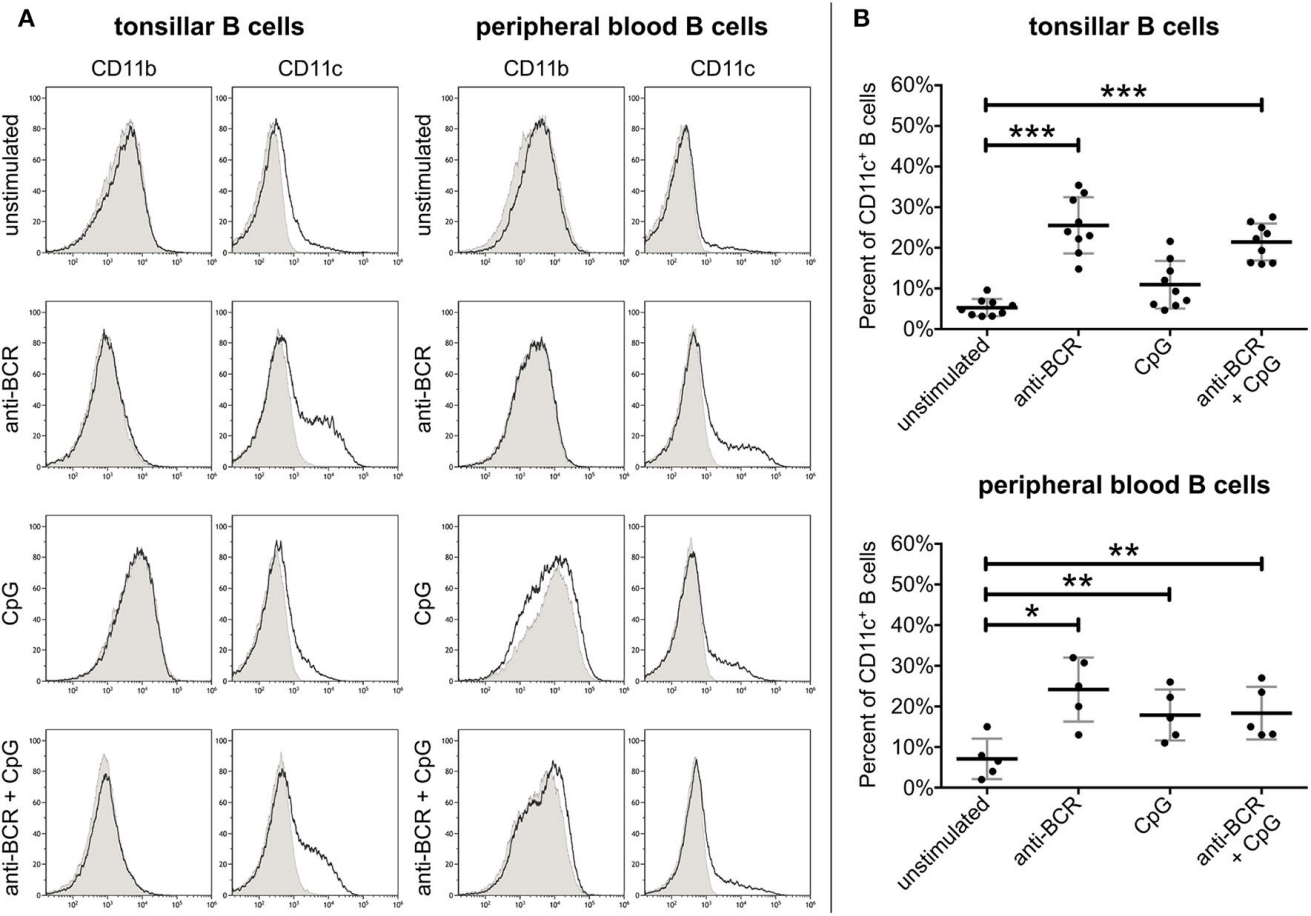
Expression of CD11b and CD11c on human B lymphocytes. CR3 and CR4 expression was measured by flow cytometry using unstimulated tonsillar and peripheral blood B lymphocytes, and cells after activation with 5 μg/ml F(ab')_2_ anti-human IgG/A/M or 0.5 μg/ml CpG either separately or simultaneously. The purity of B cells was higher than 95% in each case, as measured by CD19 positivity. To rule out dead cells from the analyses we gated on cells which were negative for DAPI staining. Histograms show the results of one representative experiment **(A)**, while the diagrams summarize the results (mean ± SD) calculated from nine independent experiments in the case of tonsillar, and five independent experiments in the case of blood-derived B lymphocytes using one-way ANOVA with Tukey's post-test (*p < 0.05; **p < 0.01; ***p < 0.001) **(B)**.

As peripheral lymphoid organs are the primary site for B cell activation and tonsils contain a wider range of various B cell populations than peripheral blood, first we decided to compare the surface expression of CD11b and CD11c on B cells of both sources by flow cytometry. Measurements were carried out after 3 days of stimulation with two physiologically relevant stimuli, namely via the BCR and TLR9. As shown in Figure 1, on resting tonsillar B cells no significant CR3, and only a slight CR4 expression was detected. After 3 days of BCR stimulation with 5 μg/ml goat anti-human IgG/A/M F(ab')_2_, up to 35% of the cells expressed CD11c. Activation with 0.5 μg/ml of CpG, the TLR9 agonist also induced CD11c expression in up to 21% of B cells, however, the average ratio of CD11c^+^ cells among TLR9 stimulated tonsillar B lymphocytes was not significantly higher than that of the non-stimulated B cells. Interestingly, the simultaneous trigger induced a lower percentage of CD11c^+^ B lymphocytes than the BCR stimulus alone. In the case of blood-derived B cells we also found that BCR stimulation was the strongest trigger to induce CD11c expression, while lower percentages of B lymphocytes were positive for CD11c after stimulation via TLR9 alone or via TLR9 and BCR combined. In contrast to CR4 however, none of the indicated stimuli induced CD11b expression on tonsillar or blood-derived B lymphocytes (Figure 1).

### Kinetics of BCR-Induced CR4 Expression and the Number of CD11c on the Surface of Activated Human B Cells

As trafficking of β_2_ integrins can be a dynamic process, we set out to define the kinetics of CD11c expression after BCR stimulation. To this end, we assessed CR4 expression at mRNA and protein level simultaneously on days 1, 2, and 3 after activation.

**Figure 2:**
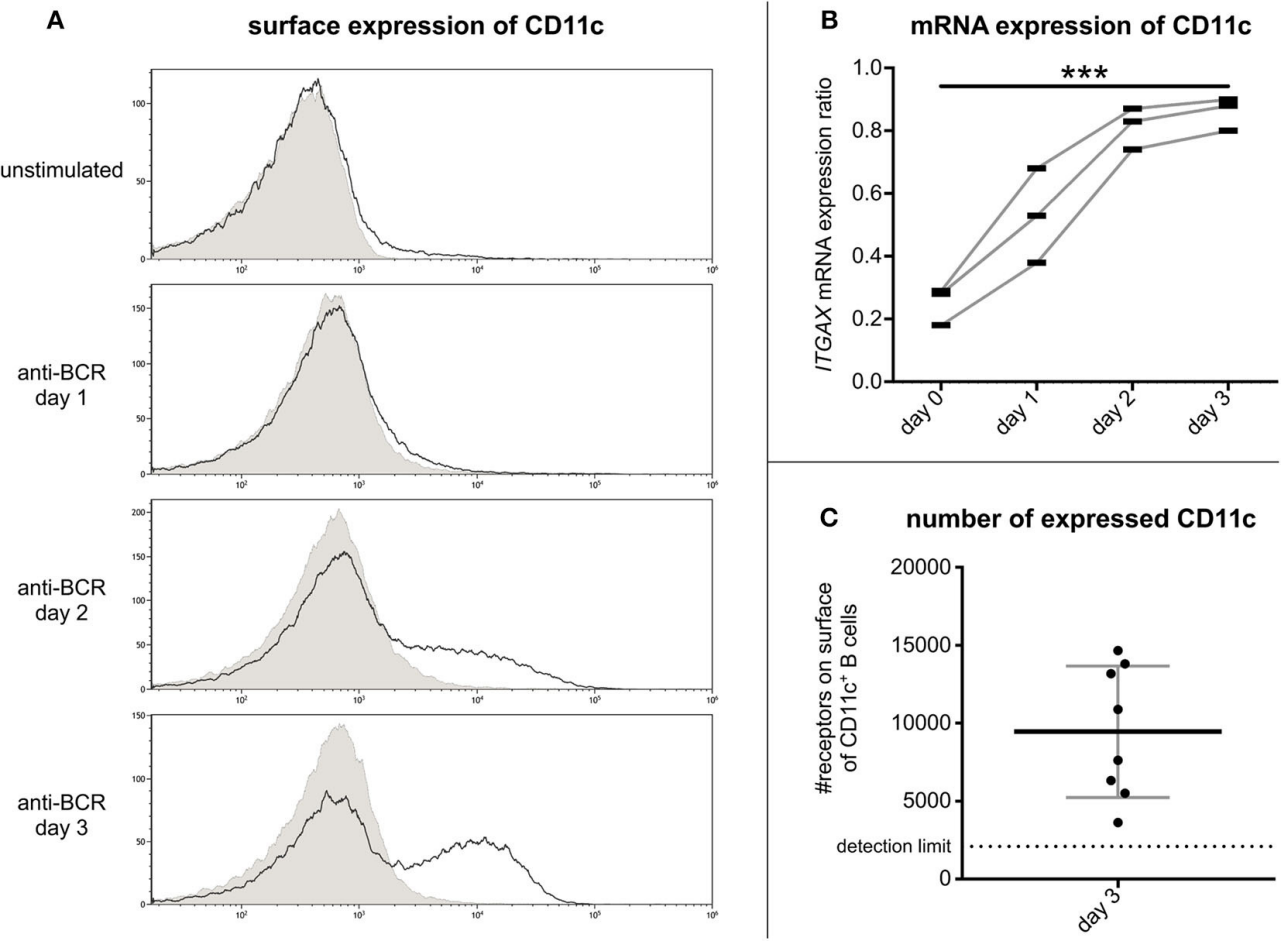
Kinetics of CR4 expression on BCR activated tonsillar B lymphocytes and the number of receptors. The surface expression of CD11c was measured by flow cytometry on resting tonsillar B cells and after 1, 2, or 3 days of activation using 5 μg/ml goat anti-human IgG/A/M F(ab')_2._ Results of one representative of three independent experiments are shown (**A**). Values of RQ-PCR analysis represent expression ratios of the CD11c coding mRNA *ITGAX* normalized to the expression level of the housekeeping gene *GUSB* (three independent experiments). We used one-way ANOVA with post-test for linear trend to test whether the values increase from left to right order (*p < 0.05; **p < 0.01; ***p < 0.001) (**B**). The number of CD11c was determined by flow cytometry using Qifikit calibration beads on tonsillar B cells 3 days after BCR induced activation (Mean ± SD was calculated from eight independent experiments) (**C**).

Flow cytometry measurements revealed that on tonsillar B lymphocytes the cell surface expression of CR4 increases on day 2 after activation and reaches its maximum on day 3 (Figure 2A). Our RQ-PCR analysis revealed that the amounts of coding mRNA for CD11c (*ITGAX*) elevates after 24 h of stimulation and reaches its maximum on the second day, i.e., 1 day before the maximum of the protein expression on the cell surface (Figure 2B). This finding implies that the CR4 molecules appearing on the B cell surface are newly synthesized. Verifying our cytometry data, no enhanced expression of the coding mRNA for CD11b (*ITGAM*) was found in the case of non-stimulated or stimulated B cell samples (data not shown).

So far, the density of CR4 molecules on human B lymphocytes has not been determined, though this information is important to better understand the role of CD11c in various B cell functions. For this reason, we assessed the number of this β_2_-integrin on day 3 after the BCR stimulus. Using the Qifikit calibration beads, we revealed that on average 9500 CD11c molecules are present on the cell surface (Figure 2C). This density is higher than that measured on monocytes (6) or neutrophil granulocytes (1), indicating a level of expression that is high enough to exert important functions of B lymphocytes.

### Activation-Induced CD11c Expression of Memory B Cells Occurs Parallel With Ig Class Switching

Rubtsov et al. characterized blood derived CD11c^+^ B lymphocytes as IgD^−^, IgM^−^, IgG^+^, CD38^low^, CD5^high^, CD80^high^, CD86^high^, CD20^high^, CD23^−^, CD27^high^ B cells (11), that are basically switched memory B cells. In a more detailed study of their classification ~62% of CD11c^+^ B cells were found to represent the switched (CD27^+^IgD^−^) and 16% to the unswitched (CD27^+^IgD^+)^ memory B cell pool. At the same time, the frequency of CD11c^+^ B cells was reduced in the naïve (CD27^−^IgD^+^) as well as in the double negative (CD27^−^IgD^−^) population. Yet, CD11c was detected in each subpopulation suggesting a role for CR4 in all steps of B-cell development (13). Since the phenotype of tonsil derived CD11c^+^ human B cells has not been defined so far, we set out to characterize this population. We also compared the distribution of CD11c^+^ B lymphocytes in the population of unstimulated and BCR activated B cells.

**Figure 3:**
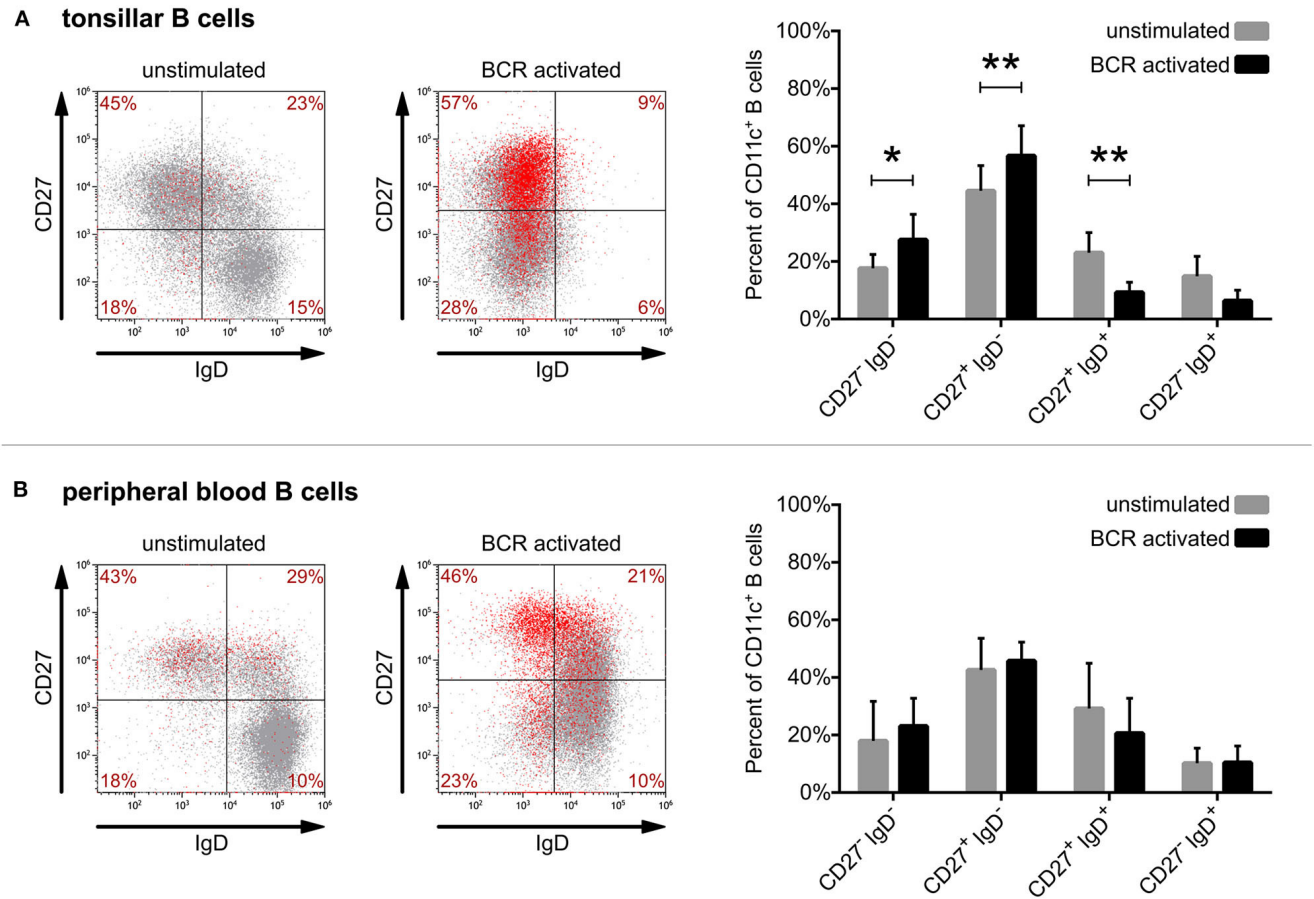
Defining the phenotype of CD11c^+^ human B cells derived from tonsil and blood. Surface expression of CD27, IgD, and CD11c was assessed by flow cytometry right after isolation (unstimulated) or after 3 days of activation with 5 μg/ml goat anti-human IgG/A/M F(ab')_2_ (BCR activated) on tonsillar (**A**) or blood-derived B cells (**B**). Dot-plots show the results of one representative experiment, where red dots represent CD11c^+^ B cells, while the distribution of CD11c^+^ cells among the 4 quadrants presented as percentages are means of 8 independent for tonsillar, and of 5 independent for blood-derived B cells. The effect of activation on the distribution of CD11c^+^ B cells (mean ± SD) was calculated from the results of 8 donors for tonsillar and of 5 donors for blood-derived B cells using two-way ANOVA with Sidak post-test (*p < 0.05; **p < 0.01; ***p < 0.001).

By triple-staining of unstimulated tonsillar B cells for CD27, IgD, and CD11c we found that the vast majority of tonsillar CD11c^+^ B cells also belong to the switched memory population (CD27^+^IgD^−^). Lower numbers of CD11c^+^ B cells were found in the unswitched memory (CD27^+^IgD^+^) population and only a few CD11c^+^ cells were detected among the double negative (CD27^−^IgD^−^) and naive (CD27^−^IgD^+^) B cells (Figure 3A). While blood and tonsils contain different subsets of B lymphocytes (e.g., blood lacks GC B cells), we found that the distribution of non-activated CD11c^+^ B cells in tonsil and blood is very similar (Figure 3B).

However, when comparing the distribution of CD11c^+^ B cells after 3 days of activation via the BCR, one can observe significant changes in the case of tonsillar B lymphocytes. While the frequency of switched memory (CD27^+^IgD^−^) as well as of double negative (CD27^−^IgD^−^) B cells increased significantly in the CD11c^+^ B cell pool, the frequency of unswitched memory B cells and naïve B cells decreased (Figure 3A). Though BCR activation induced the increase of CD11c expressing tonsillar as well as blood B cells (Figure 1), in the case of the latter no significant changes were observed regarding their distribution (Figure 3B).

Overall, our data show that while the vast majority of both tonsil and blood derived CD11c^+^ B cells belong to the switched memory pool with or without BCR stimulation, the ratio of switched memory B cells further increases among tonsillar CD11c^+^ B cells after 3 days of activation. This suggests that the activation induced CD11c expression of tonsillar B cells occurs mostly in parallel with class switching.

### Role of CR4 on BCR-Activated Tonsillar Memory B Cells

While various characteristics of CD11c^+^ blood derived B cells were described recently by Golinski et al. (13), the genuine role of CR4 expressed on B cells activated via a physiological stimulus has not been clarified yet. To this end, we decided to reveal the function of this β_2_ integrin in adhesion, proliferation, and migration on BCR activated human B lymphocytes. In preliminary experiments we confirmed that under the conditions of the functional assays β2 integrins on BCR activated CD11c^+^ B cells were switched into their active state (Supplementary Figure 1).

### CR4 Mediates Adhesion of Activated B Cells to Fibrinogen Coated Surface

**Figure 4:**
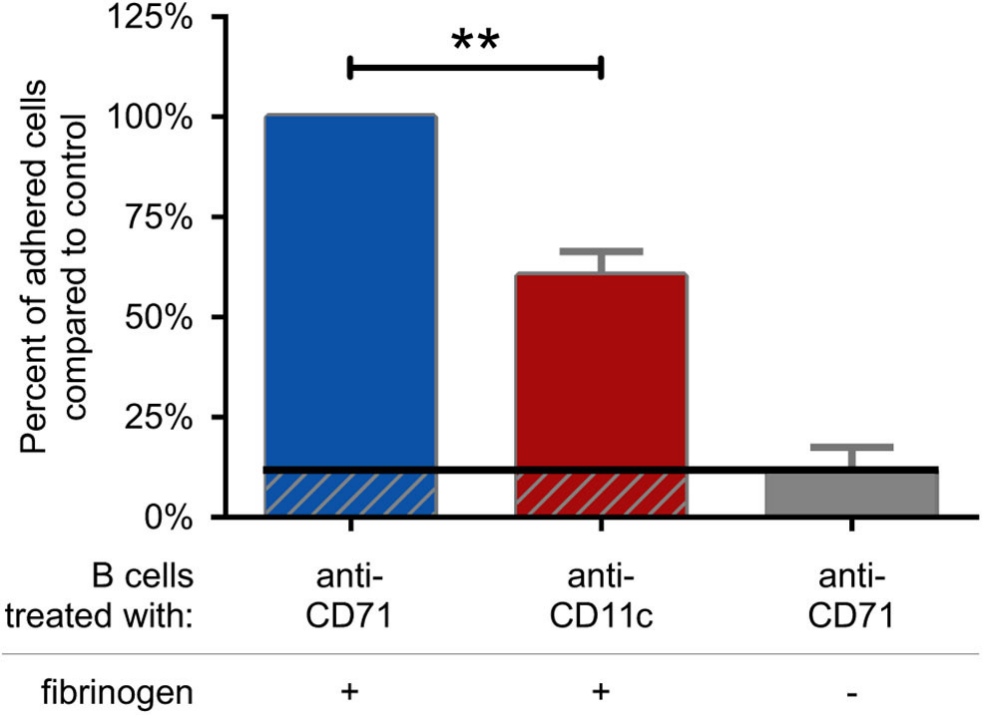
CR4 mediates adhesion of activated human B cells to fibrinogen. 5 μg/ml F(ab')_2_ anti-human IgG/A/M stimulated human tonsillar B cells were let to adhere to surfaces coated with fibrinogen and blocked by PLL-PEG (blue and red columns) or to surfaces without fibrinogen (gray column). Adherence was performed in the presence of CD71 or CD11c specific antibody. The diagram summarizes the results (mean ± SD) calculated from four independent experiments using one-way ANOVA with Tukey's post-test (*p < 0.05; **p < 0.01; ***p < 0.001).

First we set out to assess the adhesive capacity of BCR-activated cells to fibrinogen coated surface. We found, that blocking the function of CR4 with a CD11c specific antibody significantly decreased the adherence of B lymphocytes, compared to the control samples. As control, cells were treated with CD71 specific antibody, which is of the same isotype as the CD11c blocking antibody and binds to the transferrin receptor that is not involved in B cell adherence (Figure 4). Samples, where CD71 specific antibody treated cells were let to adhere to a surface without fibrinogen coat, served as negative control and in this case only a low number of adherent cells could be detected. Thus, our data demonstrate that CR4 specifically mediates adhesion of activated human B cells to fibrinogen, the natural ligand of this β_2_ integrin. To strengthen the results obtained using the CD11c blocking antibody, we also carried out the experiments employing CD11c^−^ cells only. When performing the adhesion assay using only CD11c^−^ B lymphocytes, significantly lower number of cells were able to adhere to fibrinogen under the same conditions, moreover, the adhesion could not be further blocked by the CD11c specific antibody (Supplementary Figure 2).

### CR4 Mediated Adhesion Enhances the Proliferation of B Cells

As we found that CR4 mediates the adhesion of human B lymphocytes to fibrinogen, we set out to investigate whether this process affects the proliferative capacity of the cells. We carried out the proliferation assay on fibrinogen coated surface where non-specific binding sites were blocked by PLL-PEG. As controls, we employed non-treated surface, where cells could adhere to any undefined structure, and PLL-PEG treated surface, where adhesion was hindered.

**Figure 5:**
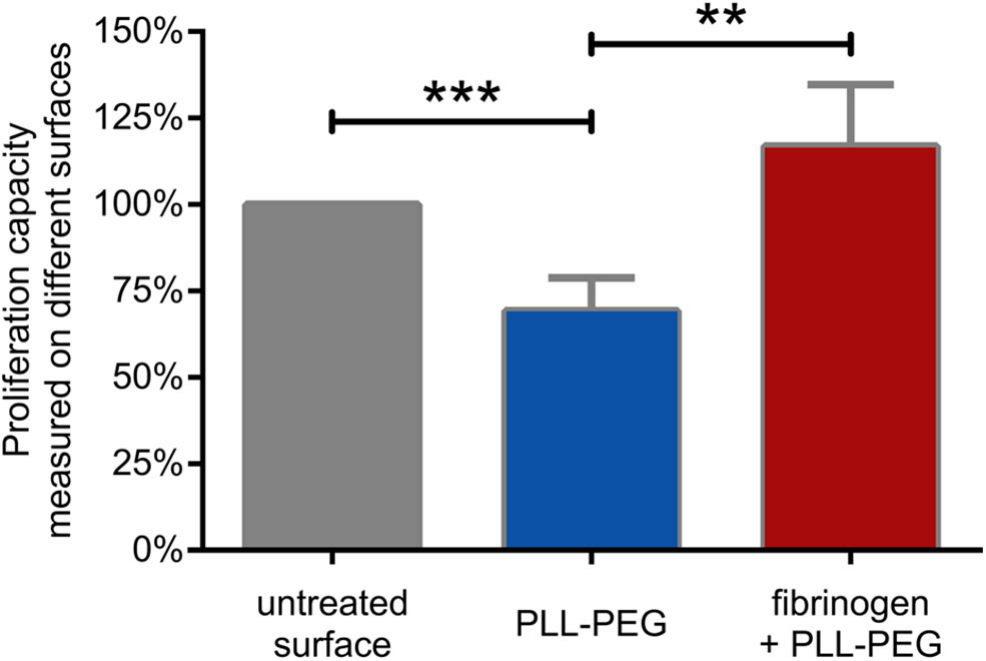
B cell proliferation is significantly enhanced by CR4 mediated adhesion. The proliferation of BCR activated cells was measured on non-treated (gray) PLL-PEG-treated (blue) or fibrinogen coated plus PLL-PEG treated (red) surface. Data are normalized to the proliferation rate measured on non-treated surface (indicated as 100%, gray column). The diagram summarizes results (mean ± SD) calculated from seven independent experiments using one-way ANOVA with Tukey's post-test (*p < 0.05; **p < 0.01; ***p < 0.001).

We found that the proliferative capacity of BCR activated cells was significantly decreased on PLL-PEG, underlining the importance of adhesion for proliferation. On fibrinogen-coated surface however, the proliferative capacity of the BCR activated lymphocytes was significantly increased, demonstrating the involvement of CR4 in this process (Figure 5).

### CR4 Contributes to the Migration of Activated B Cells

**Figure 6:**
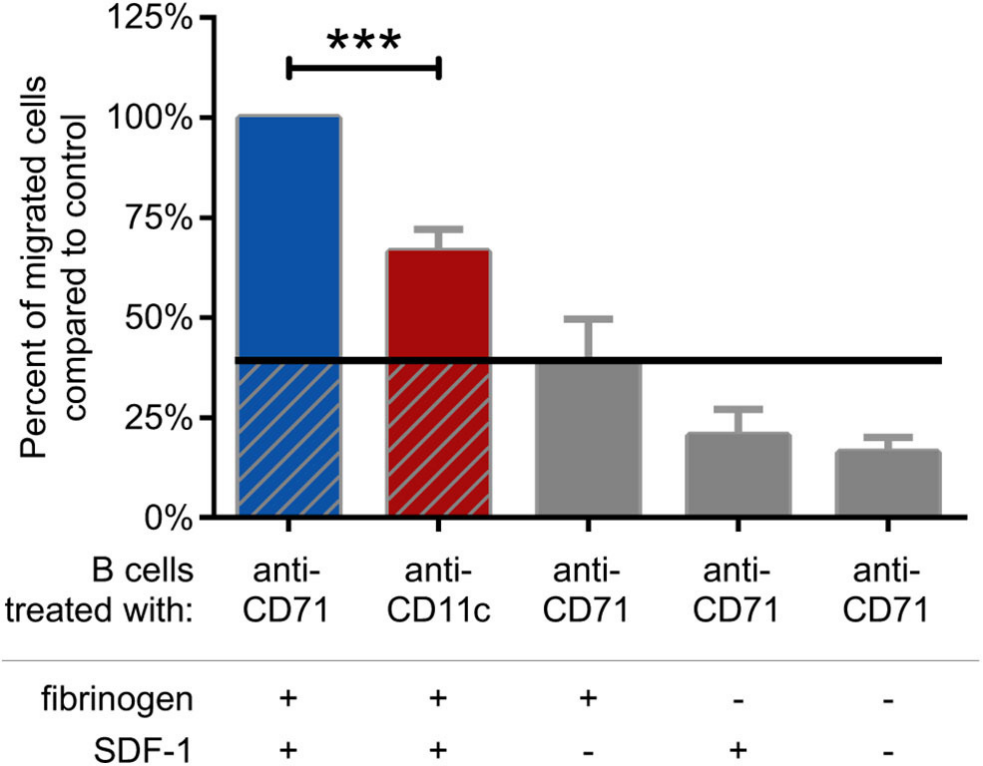
Migration of BCR stimulated cells through fibrinogen coated membrane toward SDF-1 is blocked by CD11c specific antibody. Cells were treated with CD11c-specific antibody (red), and the number of transmigrated cells were counted by flow cytometry. Results of 5 donors are shown as mean ± SD, normalized to the control sample presented as 100% (blue). As negative control CD71 specific antibody treated cells were let to migrate through PLL-PEG blocked membrane without fibrinogen and/or in the absence of the chemoattractant SDF-1 (gray columns) One-way ANOVA with Tukey's post-test was used to determine significant differences compared to control (*p < 0.05; **p < 0.01, ***p < 0.001).

Finally, we analyzed the migration capacity of BCR activated cells. We found, that blocking CR4 with a ligand binding site specific antibody significantly decreases this function, as compared to the CD71 specific antibody treated cells (Figure 6). The measurement was performed in a transwell assay using fibrinogen coated and PLL-PEG blocked membrane, in the presence of SDF-1, as chemoattractant. We used CD71 specific antibody treated cells as negative control, which were let to migrate through a PLL-PEG blocked membrane without the fibrinogen coat, and/or in the absence of the chemoattractant in the lower chamber (gray columns on Figure 6). We confirmed the results of these experiments by employing CD11c^−^ cells separately. In this case we found, that significantly lower number of cells were able to migrate under the same conditions, moreover, the migration could not be further blocked by the CD11c specific antibody (Supplementary Figure 3).

## Discussion

Complement receptors CR3 and CR4 are members of the β_2_ integrin family and have a well-known role in actin linked functions including phagocytosis, adhesion and migration. While these two receptors have earlier been thought to mediate overlapping functions, recently we have shown that they rather can be considered as “non-identical twins” (1), since CR3 has a dominant role in phagocytosis (5) while CR4 prevails over CR3 in adhesion to fibrinogen on human monocytes, macrophages and dendritic cells (6).

However, the available data regarding the appearance and certain functions of CR3 and CR4 on human B cells are rather inconsistent [reviewed in (1)]. Ehrhardt et al. showed that the expression of these receptors is mutually exclusive (16), while in a very recent study, Golinski et al. showed that the CD11b mRNA can be upregulated in CD11c^+^ B cells (13).

Previously, CR3 expression was detected on 20% of human peripheral B cells by Muto et al. (17), and Rothstein et al. identified a small subset of B-1 cells to be CR3^+^ (9), while others found no significant expression of CD11b on tonsillar or peripheral B lymphocytes, not even after activation (7, 8). So far only Kawai et al. addressed the function of this β_2_ integrin and demonstrated a high migratory potential of a CD11b^+^ blood memory B cell population (12).

Regarding CR4, Wormsley et al. found that ~20% of peripheral blood B cells express CD11c (10). In contrast, Rubtsov et al. identified only low numbers of CD11c^+^ peripheral B cells in healthy donors, which were found to be expanded in older women with autoimmune disease (11). Very recently, Golinski et al. demonstrated that the number of CD11c^+^ peripheral B cells is in correlation with the age of the healthy donors, varying between 3 and 55 percentages. They also found that CR4 expression is upregulated by stimulation via the BCR but not through TLR9 or TLR7. Furthermore, activated CD11c^+^ B cells were shown to differentiate into antibody-secreting cells (13). The questions however, whether and how CR4 participates in this process has not been addressed in this study. Much earlier, Postigo detected CR4 on phorbol myristate acetate (PMA) stimulated human B lymphocytes and showed its participation in the adhesion and proliferation of B lymphocytes (7). Since PMA is a non-physiological stimulator of the cells having a strong effect on integrins themselves (18), we deemed important to investigate in detail how the physiologically relevant BCR or TLR9 stimuli influence the expression and function of CR4.

We found that after stimulation via the BCR ~26% of tonsillar B lymphocytes expressed CD11c, while CD11b could not be detected neither at mRNA, nor at protein level. TLR9 stimulation resulted in CD11c expression in about 11% of the B cells, and after the simultaneous trigger via the BCR and TLR9 we detected CR4 in 21% of B lymphocytes on average (Figure 1). Golinski et al. also found that stimulation via the BCR leads to the upregulation of CR4 in B cells, this however could not be induced by the ligation of TLR7 and TLR9 (13). On the other hand, Rubtsov et al. described that the accumulation of CD11c^+^ B cells in mice is driven by TLR7 (11). While these data might suggest a differential regulation of CD11c expression in B cells of men and mice, the contradictory results could also be ascribed to the different methods applied. In our study we found, that not only the BCR, but also the TLR9 mediated activation increased the number of CD11c^+^ B cells, which also seems to contradict the results of Golinski et al. However, Golinski et al. tested if CD11c^−^ cells can be induced to express the receptor, while we stimulated the whole B cell pool. Therefore, it is possible, that in our experimental conditions the increase in the number of CR4^+^ B cells was due to the TLR9-induced proliferation of CD11c^+^ cells.

Nevertheless, with BCR stimulation being clearly the strongest physiological trigger to induce CD11c expression, we analyzed the kinetics of the expression and the function of CD11c on human B lymphocytes activated through their antigen binding receptors. We found that CD11c molecules appearing on the B cell surface are newly synthesized and reach maximal expression level on day 3 (Figures 2A,B). Assessing the number of these molecules on activated B cells we identified 9,500 receptors per cell on the average (Figure 2C). This is a higher density than that measured on monocytes (6) or on neutrophil granulocytes (1), highlighting the importance of CR4 on B cells.

Regarding the phenotype of CD11c^+^ human B lymphocytes we found that the vast majority of tonsillar B cells also belong to different memory B cell subsets, which is in line with earlier results obtained with peripheral B cells (11, 13). It is likely, that the lower number of CD11c^+^ cells among the TLR9 stimulated B lymphocytes is due to the fact that after 3 days of stimulation with the TLR9 agonist, more B cells differentiate into CD11c^−^ plasmablasts and plasma cells, and consequently the ratio of memory B cells is lower in this population than among BCR stimulated cells. The distribution of CD11c^+^ cells in the different B cell subsets among TLR9 stimulated B lymphocytes is an interesting field of research in the future, but in the present study, we focused on the distribution of BCR stimulated CD11c^+^ B cells. We demonstrated, that the increase of CD11c expressing cells among BCR activated tonsillar lymphocytes occurs in parallel with Ig class switching, as tonsillar CD11c^+^ B cells contain significantly higher number of switched and significantly lower number of unswitched memory B cells after 3 days of stimulation (Figure 3). Yet, it needs to be answered whether these CD11c^+^ cells are newly generated, or reactivated memory B lymphocytes and whether they utilize CR4 in order to differentiate into memory B cells, or mature memory B cells use it during their latter functions. To answer these questions, we need further studies on the processes during which memory B cells are generated and reactivated.

It has been known for long that most memory B cells are formed in germinal centers (GCs). Newly generated memory B cells might either migrate to different sites throughout the body under the guidance of chemokine receptors, or they can be re-activated to proliferate and generate more memory B cells before terminal differentiation into antibody-secreting plasma cells (19). Regarding the place of re-activation it is known, that memory B cells not only originate from the GCs, but they are able to re-enter the GC reaction to proliferate and further diversify their BCRs at later time points (20–22) possibly even during the primary infection (19).

Based on our results we raised the question, whether CR4 of activated B cells could play a role in this process? As one of the major natural ligands of this β_2_ integrin is fibrinogen, CR4 can be assumed to be involved in the adhesion and migration of B cells where fibrinogen is involved. This presumption is reinforced by the findings of Lefevre et al., demonstrating high amounts of fibrinogen on the surface of follicular dendritic cells (FDC) in the dark zone of GCs of human tonsils (15), where both the generation and re-activation of memory B cells take place. In this paper the authors proposed that “further studies will be required to identify the discrete B cell population present in GC, which responds to fibrinogen.” Our data clearly prove that CD11c^+^ B cells use CR4 to adhere to fibrinogen (Figure 4), thereby may represent this fibrinogen-responsive B cell population.

Lefevre et al. also showed, that the presence of fibrinogen stimulates the proliferation and survival of the BCR stimulated L3055 cell line, which represents a clonal population of centroblasts (15). Here we show that binding to fibrinogen enhances the BCR induced proliferation of the primary, tonsil-derived B lymphocytes as well. We found, that while blocking the adhesion hampered the proliferation of tonsillar B cells, CR4 mediated adhesion to fibrinogen significantly enhanced this important process (Figure 5).

This phenomenon is very likely to occur *in vivo* within human tonsils when B cells are activated by antigen and form GCs in close contact with the FDC network. FDCs play a key role in maintaining immunological memory by presenting intact antigens to B cells (via their Fcγ receptors and complement receptor type 1 and type 2) and have been shown to support B cell survival and proliferation via different mechanisms (23). Our results provide a proof that CR4 expressed by activated B cells can contribute to establish a close contact with the fibrinogen-covered FDCs, thus facilitating the binding of antigens by B cells and allowing them to receive survival and proliferation signals. Moreover, we suggest, that adhesion to fibrinogen by CR4 itself could be one of the supporting signals B cells receive from FDCs. This hypothesis is supported by earlier findings in the case of other integrins. Namely, LFA-1 and VLA-4 were shown to be involved in the adhesion of tonsillar B cells to FDCs (24), and LFA-1 was demonstrated to facilitate the synapse formation of B cells, thereby lowering the threshold of B cell activation (25). As a feedback, BCR stimulation was shown to induce both the LFA-1 (26) and VLA-4 mediated adhesion (27) of B cells.

It is important to point out that while the light zone of GCs is rich in FDCs, the fibrinogen-covered FDCs were found only in the dark zone (15), which is the primary site of intense B cell proliferation and somatic hypermutation (28). B cell access to the dark zone is dynamically regulated by the expression of CXCR4 (29), driving B lymphocytes toward the chemoattractant SDF-1, produced by the reticular cells of the dark zone (30). Our present data showing that CR4 is involved in the migration of activated B cells toward SDF-1 (Figure 6) give a strong support to the important role of this β_2_ integrin in the GC reactions. Another scenario might be, that CD11c^+^ B cells, as they differentiate into antibody-secreting cells upon activation (13), use CR4 during their homing into the bone marrow, where SDF-1 is also expressed (31). As CD11c is known to be expressed in various B cell malignancies (8, 32), it is also very likely, that CR4 has an important role in the pathomechanism of these diseases. Interestingly, in many of these pathological cases CR3 was shown to be expressed together with CR4 (8, 33, 34).

In conclusion, we have shown that CR4 contributes to the adhesion and migration of human B cells, thereby being involved in the activation, proliferation and differentiation, as well as in the generation or re-activation of memory B lymphocytes. We suggest that this β_2_ integrin plays an important role in GC reactions by helping B cells to migrate into the dark zone and establish a close contact with fibrinogen covered FDCs, which retain native antigens on their surface. We postulate that this multipoint attachment between the two cells elevates the proliferation and affinity maturation of antigen specific memory B lymphocytes in GCs, *in vivo*.

## Data Availability Statement

The raw data supporting the conclusions of this article will be made available by the authors, without undue reservation.

## Ethics Statement

The studies involving human participants were reviewed and approved by Ethics Committee of the Medical Research Council in Hungary (TUKEB), 52088/2015/EKU. Written informed consent to participate in this study was provided by the participants' legal guardian/next of kin.

## Author Contributions

ZN-B, AE, and ZB designed the study. ZN-B, AM, and RK performed the experiments. ZN-B and RK interpreted the results. ZN-B, ZB, CB, and AE prepared the manuscript. AE supervised the research. All authors contributed to the article and approved the submitted version.

## Conflict of Interest

The authors declare that the research was conducted in the absence of any commercial or financial relationships that could be construed as a potential conflict of interest.
