# Supplementary figures and images for: Activated Human Memory B Lymphocytes Use CR4 (CD11c/CD18) for Adhesion, Migration, and Proliferation
Source: Front Immunol. 2020 Sep 29;11:565458. doi: 10.3389/fimmu.2020.565458 (PMC7550640; doi:10.3389/fimmu.2020.565458)

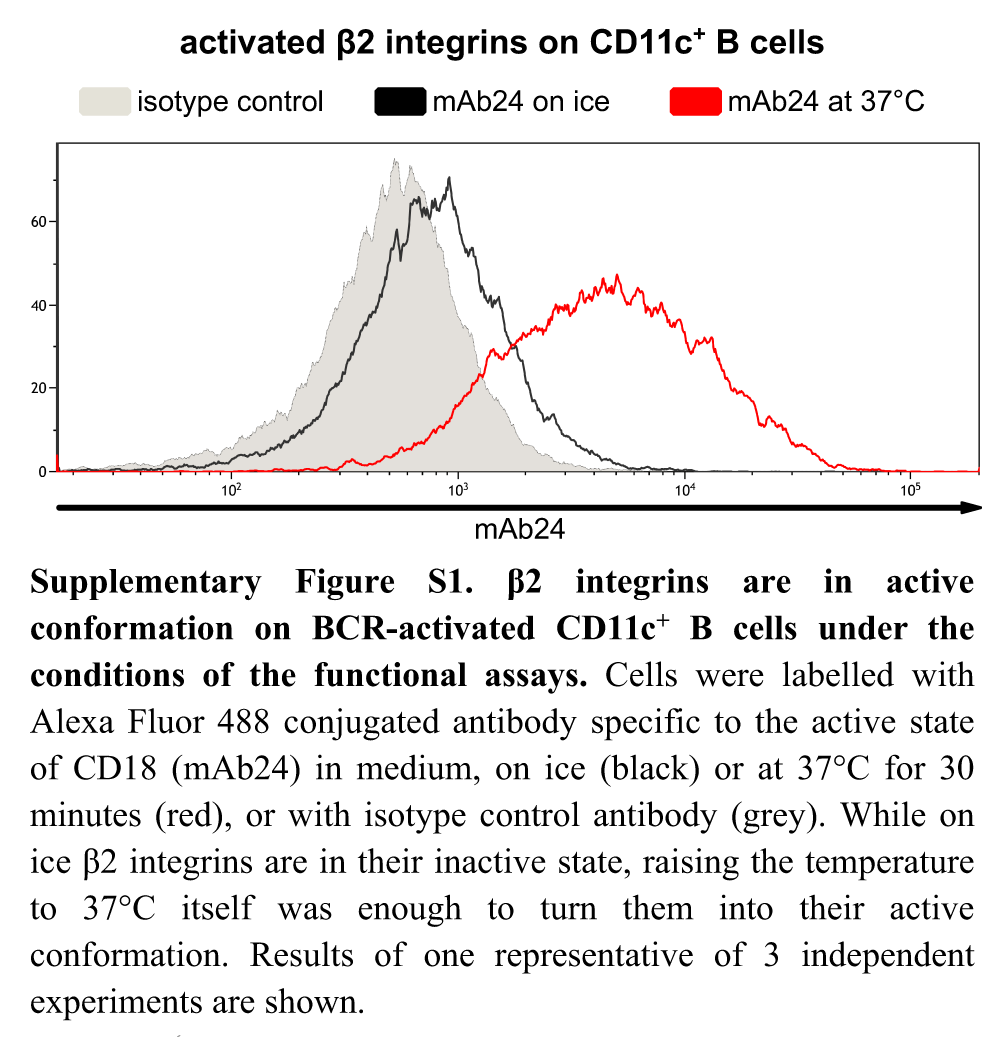

Supplement: Supplementary file 1 [file Image_1.TIF]

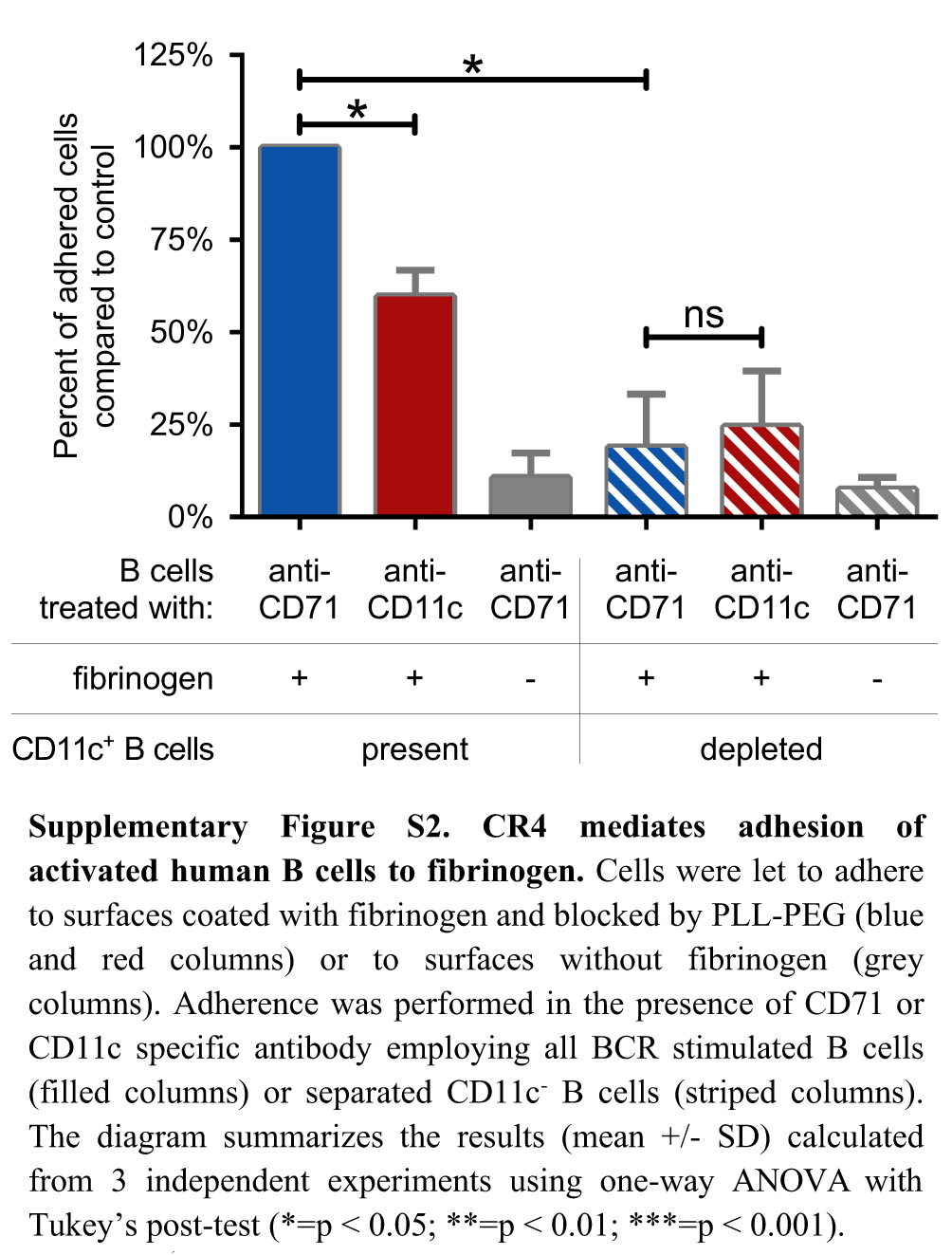

Supplement: Supplementary file 2 [file Image_2.TIF]

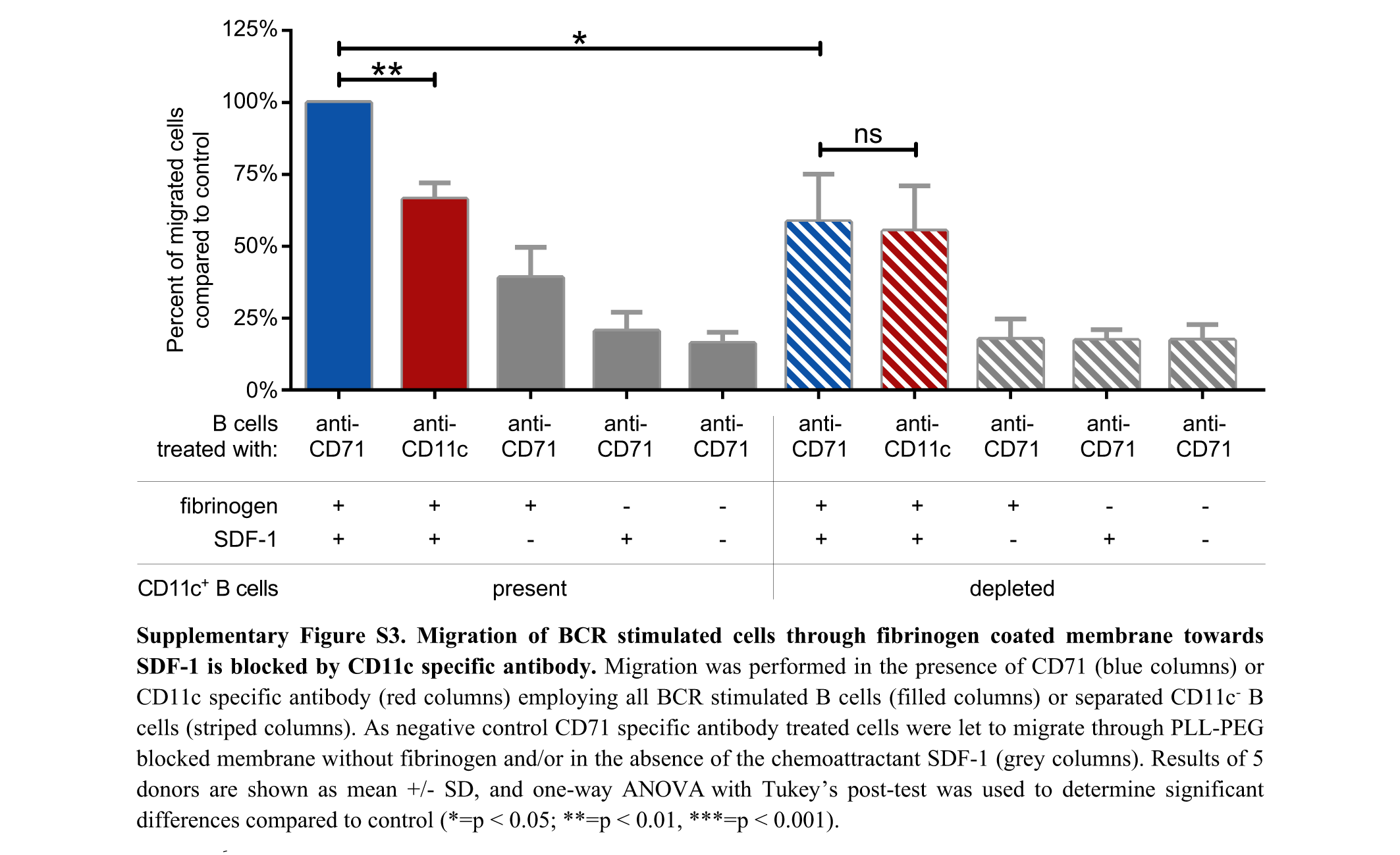

Supplement: Supplementary file 3 [file Image_3.TIF]
